# Tumor gene expression signatures associated with outcome in large B−cell lymphoma treated with CD19-directed CAR T−cell therapy (axicabtagene ciloleucel)

**DOI:** 10.3389/fonc.2025.1519473

**Published:** 2025-02-27

**Authors:** Yuan Tian, Justin Budka, Frederick L. Locke, Jason R. Westin, Christina To, Gayatri Tiwari, Daqin Mao, Davide Bedognetti, Rhine R. Shen, Jorge Andrade, Simone Filosto

**Affiliations:** ^1^ Kite, a Gilead Company, Santa Monica, CA, United States; ^2^ Department of Blood and Marrow Transplant and Cellular Immunotherapy, Moffitt Cancer Center, Tampa, FL, United States; ^3^ Department of Lymphoma and Myeloma, The University of Texas MD Anderson Cancer Center, Houston, TX, United States

**Keywords:** CAR T cells, large B cell lymphoma, axicabtagene ciloleucel, transcriptomics, gene expression, tumor biopsies, CD19

## Abstract

**Introduction:**

CAR T cell therapy provided transformative outcomes for patients with B-cell lymphoma; however, a large fraction of patients remains at risk for relapse, underlying the need to uncover mechanisms of resistance and predictive biomarkers. Herein, we leveraged the ZUMA-7 phase III randomized trial of relapsed/refractory large B-cell lymphoma (LBCL) patients treated with axicabtagene ciloleucel (axi-cel; CD19-targeting CAR T cells) to discover tumor gene expression signatures (GES) associated with outcome.

**Methods:**

With tumor transcriptomics from 134 axi-cel patients, we employed multivariate penalized Cox models analyzing event-free survival (EFS), progression-free survival (PFS), and duration of response (DOR).

**Results and Discussion:**

We identified two novel GES, a six-gene/transcript signature (6-GES; CD19, CD45RA, CCL22, KLRK1, SOX11, SIGLEC5) correlated with improved outcome after axi-cel (HR: 0.27, 95% CI: 0.16–0.44 for EFS), representing lymphomas with abundant target antigen (CD19) expression, adhesion molecules, and relatively low immune infiltration mostly composed of cytotoxic lymphocytes (T and NK cells) and DCs, and secondly, a 17-gene/transcript signature (17-GES; CD45RO, BCL2, IL-18R1, TNFSF4 [OX40L], KLRB1 [CD161], KIR3DL2, ITGB8, DUSP5, GPC4, PSMB5, RPS6KB1, SERPINA9, NBN,GLUD1, ESR1, ARID1A, and SLC16A1) correlated with disease progression after axi-cel (HR: 6.12, 95% CI: 3.57–10.50 for EFS), consistent with high immune inflammation and escape mechanisms, such as the upregulation of genes involved in repair of damaged DNA or chromatin remodeling, inhibition of apoptosis, and a metabolically restrictive environment. These signatures did not correlate with outcome in the standard-of-care arm of ZUMA-7 (chemotherapy, followed by transplant) or frontline therapy, supporting their predictive rather than prognostic value. The findings were technically reproduced in a subset of ZUMA-7 samples profiled by RNA-seq (axi-cel, n=124; SOC, n=125). The 6-GES was reduced, whereas the 17-GES was elevated at progression post axi-cel, consistent with the notion that these signatures represent features relevant for response and resistance to CAR T-cell therapy.

**Conclusion:**

Our transcriptomic analysis identified gene expression signatures potentially predictive of outcome with CD19-directed CAR T-cell therapy, and these findings are informative for risk stratification and development of next-generation products.

## Introduction

Axicabtagene ciloleucel (axi-cel) is an autologous anti-CD19 chimeric antigen receptor (CAR) T-cell therapy approved for the treatment of adults with relapsed/refractory (R/R) large B-cell lymphoma (LBCL) after two or more lines of systemic therapy and for patients refractory to, or who relapsed within 12 months of, first-line chemoimmunotherapy. In the Phase 3 ZUMA-7 study (NCT03391466), axi-cel demonstrated superior event-free survival (EFS) versus the standard-of-care (SOC) arm, consisting of platinum-based chemotherapy followed by high-dose chemotherapy and autologous stem cell transplantation in responding patients, resulting in approval of axi-cel as a second-line treatment for R/R LBCL (1). With a median follow-up of 47.2 months, axi-cel demonstrated significantly longer overall survival versus SOC, establishing axi-cel as a superior curative intent therapy (2).

Despite the transformative benefits of axi-cel therapy, approximately 60% of LBCL patients do not respond or experience disease progression after an initial response to therapy. There is an unmet need to uncover mechanisms of resistance and associated predictive biomarkers that would enable risk stratification and inform design of next-generation CAR T-cell therapy. Recently, gene expression profiling of tumors has shown great promise for the identification of factors impacting CAR T-cell therapy in R/R LBCL (3). Specifically, leveraging the NanoString IO-360 panel, as well as immune-histochemistry (IHC), we have reported that tumor target antigen (CD19) expression and a predefined B-cell signature (from NanoString) associate with longer EFS with axi-cel in R/R LBCL. Conversely, the conventional prognostic biomarker of molecular subgrouping by tumor cell of origin (GCB vs. non-GCB status) did not associate with EFS following axi-cel treatment (3).

In this study, we performed gene expression profiling on tumor samples from ZUMA-7 to identify novel and robust signatures specifically associated with outcome to axi-cel therapy. Through penalized multivariate modeling of clinical outcomes including event-free survival (EFS), progression-free survival (PFS), and duration of response (DOR), we identified two signatures that are putatively predictive of efficacy following axi-cel therapy. These two signatures outperformed all previously identified tumor biomarkers, including CD19 expression, individual genes, and B-cell signature, and provide biological insights into factors influencing CAR T-cell outcome, which could be informative for risk stratification and the development of next-generation CAR T-cell products.

## Methods

### ZUMA-7 trial design, oversight, endpoints, and assessments

The ZUMA-7 trial was conducted at 77 sites worldwide. Eligible patients were at least 18 years of age and had histologically confirmed large B-cell lymphoma, according to the World Health Organization 2016 classification criteria, that was refractory to first-line treatment or that had relapsed from complete remission no more than 12 months after the completion of first-line chemoimmunotherapy; patients intended to proceed to high-dose chemotherapy with autologous stem-cell transplantation. Refractory disease was defined as a lack of complete response to first-line therapy, and relapsed disease occurring no more than 12 months after the completion of first-line therapy.

All the patients provided written informed consent. The trial was conducted in compliance with the principles of the Declaration of Helsinki. Full details around trial design and oversight were previously reported (1, 2).

The primary end point was event-free survival (defined as the time from randomization to the earliest date of disease progression according to the Lugano classification, the commencement of new therapy for lymphoma, death from any cause, or a best response of stable disease up to and including the response on the day 150 assessment after randomization) according to blinded central review. Secondary end points included progression-free survival (defined as the time from randomization to disease progression or death from any cause), assessed centrally or by investigator and overall survival, durability of response (defined as the time between the first objective response to disease progression and the start of new lymphoma therapy), and the incidence of adverse events, as previously reported (1, 2, 4).

Endpoints utilized for the analyses reported in this manuscript are EFS, PFS, and DOR per central review from the primary EFS analysis data cut, as well as PFS per investigator assessment (as indicated in the Result section and figures) from primary overall survival analysis, which occurred 5 years after the first subject was randomized, previously published (1, 2).

Classification of patients into molecular subtypes of cell of origin (GCB vs. non-GCB), double/triple hit [high-grade B-cell lymphoma (HGBL)], MYC rearrangement, and double-expressor lymphomas (overexpression of MYC and BCL2 proteins) was based on central laboratory analysis and previously reported (1, 3).

### Multivariate analyses and discovery of gene expression signatures

Details regarding ethics, patient samples, and efficacy endpoints were described previously (3). In this *post hoc* analysis, evaluable samples collected pretreatment (axi-cel, n=134; SOC, n=122) or at progression, post axi-cel treatment (n=17), from patients in ZUMA-7 study were analyzed. Gene expression profiling was performed using the NanoString nCounter^®^ PanCancer IO 360™ Panel. RNA extraction and processing into the nCounter platform for the NanoString IO360 assay was conducted at NeoGenomics. Nanostring RCC and RLF files were imported on nSolver Analysis software (v.4.0). Raw data were further analyzed with nCounter Advanced Analysis (v.2.0.134), and normalized linear count output were used for all further analysis, as previously described (3). Predefined IO-360 NanoString signatures were also analyzed.

To identify genes whose transcripts were associated with clinical outcomes including duration of response (DOR), event-free survival (EFS), and progression-free survival (PFS), penalized Cox proportional hazards models were utilized (5). These multivariate models consider the expression of multiple genes simultaneously to select key predictors while preventing overfitting. The elastic net penalty was used to combine the feature selection capability of Lasso (L1 penalty) with the stability of Ridge (L2 penalty), improving the robustness of biomarker identification, as per below formula:


$$\arg\underset{\text{β}}{\max}\log\text{PL}\left(\text{β}\right)-\text{α}\left(r\sum_{j=1}^{p}\left|{\text{β}}_{j}\right|+\frac{1-r}{2}\sum_{j=1}^{p}{\text{β}}_{j}^{2}\right)$$

where γ was set to 0.9, and the optimal α was determined using fivefold cross validation based on concordance index (C-index), measuring predictive accuracy. Models were fitted individually for three clinical outcomes (DOR, EFS, PFS), aiming to identify overlapping transcripts to ensure a robust final signature. Non-zero coefficients were identified from the best-performing model to derive gene signatures for each clinical outcome. Transcripts/genes with negative coefficients (hazard ratio [HR]<1) were termed favorable transcripts (associated with improved outcome), whereas genes with positive coefficients (HR >1) were termed unfavorable transcripts (associated with worse outcome).

### Signature scoring and patient groups

A gene expression signatures (GES) score was assigned to each sample using the mean scaled gene expression with the following formula:


$$score=\;\frac{1}{m}\sum_{i=1}^{m}\frac{\left(Count_{i}-u_{i}\right)}{\sigma_{i}}$$

where m is the number of genes within a specific signature, $Count_{i}$ is the normalized linear count from NanoString or linear TPM value when derived from RNA-Seq (see below about technical replication) associated with $gene_{i}$ within the given signature for the given sample, $u_{i}\;$ is the mean normalized count or TPM value calculated from all samples for $gene_{i}$ , and $\sigma_{i}$ is the standard deviation of normalized count or TPM values derived from all samples for $gene_{i}$ .

The patients were subsequently divided into two groups based on their scores relative to the median. Those with scores above the median were placed in the “high” group, whereas those with scores at or below the median were assigned to the “low” group. This median split method ensures an even distribution of participants across the two groups, facilitating a balanced comparison in subsequent analyses.

### Technical replication with RNA sequencing

The predictive value of the identified signatures was replicated using a subset of ZUMA-7 samples profiled by RNA sequencing (RNA-Seq; axi-cel, n=124; SOC, n=125). Most patient samples overlapped between the NanoString gene expression and RNA-Seq analysis conducted herein (axi-cel, n=119; SOC, n=115).

Gene expression profiling was carried out from FFPE tumor samples by performing next-generation sequencing (NGS). Total RNA libraries were prepared using Illumina Stranded Total RNA Prep, Ligation with Ribo-Zero Plus kit (catalogue # 20040529). This kit utilizes the enzymatic rRNA depletion and ligation-based addition of adapters and indexes for whole transcriptome libraries, capturing coding and non-coding RNAs.

Following Illumina’s library prep protocol, 100 ng or 10 ng of total RNA, depending on the available amount, was used for rRNA depletion followed by fragmentation, cDNA synthesis, and library amplification. Resulting libraries were quantified using Qubit, and quality control was performed on TapeStation 4150 (Agilent). All libraries were diluted to equimolar concentration and pooled for sequencing on Illumina’s NovaSeq 6000 with a read length of 101-bp paired end. The BBDuk tool (6) was applied to trim the adapters and the low-quality reads. Pair-ended RNA-seq reads were mapped and quantified using Salmon v1.9.0 against the GENCODE V43 human transcriptome (https://ftp.ebi.ac.uk/pub/databases/gencode/Gencode_human/release_43/gencode.v43.transcripts.fa.gz). Gene-level counts and transcripts per million (TPM) values were generated using Tximeta v1.16.1. TPM values were used for downstream analysis. A total of 311 samples had available transcriptional data from either screening or post-infusion time point. RNA-sequencing quality control filtering was applied, removing samples with ≤10% reads mapped to coding sequences, leaving 296 samples, 277 pre-infusion samples, and 19 post-infusion samples across both arms of ZUMA-7 (axi-cel and SOC). Samples from patients who were not part of the safety subset (patients enrolled in the study but not treated) were excluded, resulting in 266 evaluable pre-infusion samples. To remove duplicated subjects, for those with multiple samples collected prior to axi-cel treatment, the sample with the latest collection date was kept. If collection dates were the same, the sample with the lower Molecular Batch ID (sample analyzed first) was retained. After duplicate removal, 249 pre-infusion samples remained available for the downstream analysis (124 from the axi-cel arm and 125 from the SOC arm of ZUMA-7).

### Analysis of first-line therapy (online datasets)

To determine the association between the signatures identified in this study and outcome to first-line R-CHOP or -CHOP-like treatment, we utilized two publicly available datasets (7, 8). The dataset from Schmitz et al. (8) included 229 patients who received immunochemotherapy (R-CHOP or R-CHOP-like therapy), whereas 522 patients who received R-CHOP as initial therapy were selected from the Reddy et al. dataset (7). The linear-scale TPM from the online dataset were scored into signature following the same formula/method described above. The available clinical outcome readout from the literature was PFS.

### Univariate analysis of gene expression signatures versus outcome

For this analysis, the *RegParallel* (version 1.12.0) R package (https://github.com/kevinblighe/RegParallel) was utilized to examine how the expression of genes influenced the rate of progression-free survival. The function *RegParallel* was run to fit the Cox proportional hazards regression model to gene expression to independently test the association between survival time and each gene. The output was analyzed with TIBCO Spotfire (version 11.4.3).

### Statistics

All analyses described herein were exploratory and retrospective, performed without adjustment for possible confounding factors. Log−rank test was used to determine statistical significance between the high and low groups in time-to-event analyses presented as KM curves, where high means > median value and low means ≤ median value. When further subgrouping was performed (e.g., patient segregated by the GES median threshold and further into GCB and non-GCB subsets), the median GES values were calculated from the entire dataset. Where indicated, an unstratified Cox regression model was used to provide an estimate of the hazard ratio and associated P-value. Wilcoxon rank-sum test was used when comparing two groups in categorical analysis. Spearman’s rank-order correlation was used to evaluate the association between variables. For these *post hoc* analyses, all P values were descriptive and P < 0.05 was considered significant. No adjustments for multiplicity testing were performed.

## Results

### Discovery of novel gene expression signatures associated with efficacy following Axi-Cel treatment

Transcriptomic analysis, generated via Nanostring nCounter technology, aimed to identify robust gene expression signatures predictive of three clinical outcomes, duration of response (DOR), event-free survival (EFS), and progression-free survival (PFS), following second-line axi-cel therapy. Penalized Cox regression models were fitted individually for each of the three endpoints to identify gene transcripts with non-zero coefficients (see methods).

**Figure 1:**
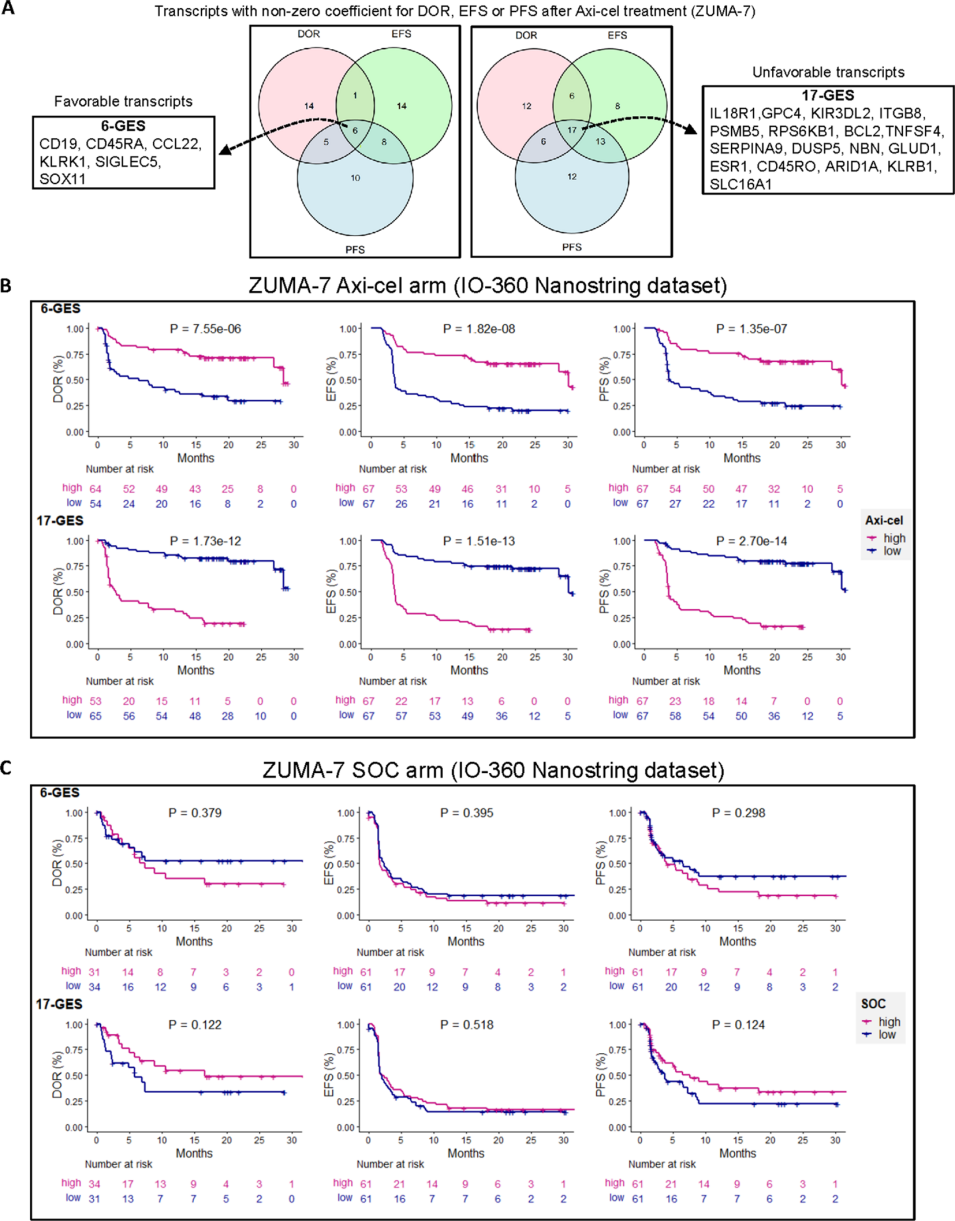
Multivariable penalized cox regression modeling led to discovery of gene expression signatures (GESs) associated with clinical outcomes in patients treated with second-line axicabtagene ciloleucel (axi-cel). **(A)** A six-gene expression signature (6-GES) was composed of CD19, CD45RA, CCL22, KLRK1, SIGLEC5, and SOX11 transcripts, which were all transcripts with negative coefficients for DOR, EFS, and PFS, corresponding to a hazard ratio less than 1 (better outcome = favorable transcripts). Conversely, a 17-GES was composed of IL18R1, GPC4, KIR3DL2, ITGB8, PSMBP5, RPS6KB1, BCL2, TNFSF4, SERPINA9, DUSP5, NBN, GLUD1, ESR1, CD45RO, ARID1A, KLRB1, and SLC16A1, which were all transcripts with positive coefficients for DOR, EFS, and PFS (hazard ratio > 1; worse outcome = unfavorable transcripts). B and C) Kaplan–Meier curves showing DOR, EFS, and PFS (per central review) stratified by high or low GES score (high GES, > median; low GES, ≤ median) in axi-cel **(B)** or SOC **(C)** arms of ZUMA-7, as indicated. P-values from log-rank tests compare the survival distributions between high and low GES groups.

Such analysis identified two distinct gene transcript lists, encompassing transcripts favorably or unfavorably linked to the three clinical outcomes (DOR, EFS, and PFS) in patients receiving axi-cel, as represented in **Figure 1A**. The full list of transcripts with the non-zero coefficient for each outcome is provided in **Supplementary Table 1**, split into two tabs with favorable or unfavorable transcripts. A list of six transcripts, herein referred to as 6-GES, representing the intersection of favorable transcripts for each outcome included CD19, CD45RA, CCL22, KLRK1, SIGLEC5, and SOX11. Conversely, a list of 17 transcripts, herein referred to as 17-GES, representing the intersection of unfavorable transcripts, included IL18R1, GPC4, KIR3DL2, ITGB8, PSMB5, RPS6KB1, BCL2, TNFSF4, SERPINA9, DUSP5, NBN, GLUD1, ESR1, CD45RO, ARID1A, KLRB1, and SLC16A1.

The gene expression values of the transcripts included in these two lists (6-GES or 17-GES) were then used to generate a scaled mean signature value of either 6-GES or 17-GES for each patient.

**Figure 2:**
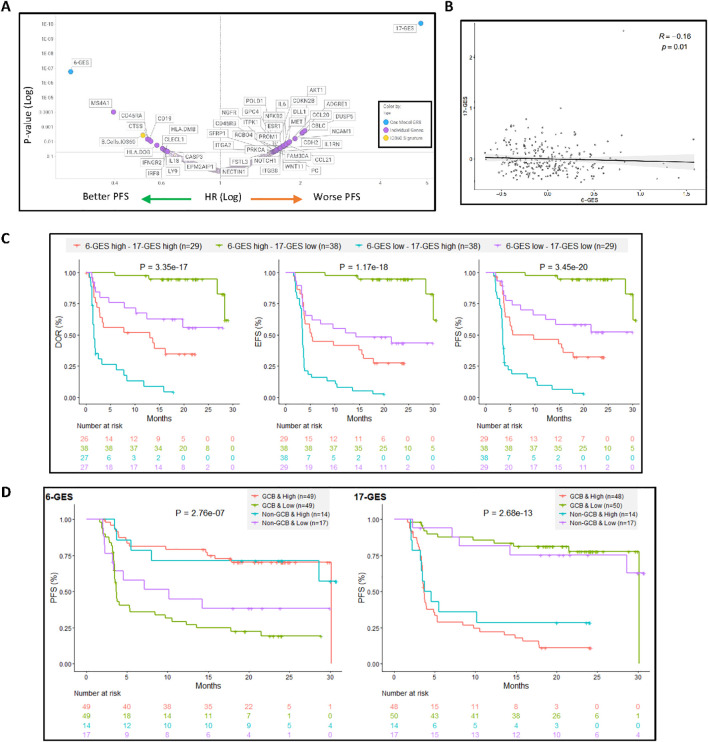
6-GES and the 17-GES outperformed all the individual gene transcripts and all predefined GES from NanoString for their association with PFS following axi-cel treatment and are relevant in both GCB and non-GCB subgroups. **(A)** Volcano plot showing the 6-GES, 17-GES, NanoString IO360 Signatures, and individual transcripts associated with PFS (per investigator) in the axi-cel arm of ZUMA-7. P values and hazard ratios (HR) were calculated via a Cox proportional hazards model, where patients were divided into two groups based on biomarker median value split. P-value and HR for each biomarker are shown. **(B)** Spearman correlation between 6-GES and 17-GES. **(C)** The combined impact of 6-GES and 17-GES on clinical outcomes in axi-cel-treated patients is shown by Kaplan–Meier curves depicting DOR, EFS, and PFS (per central review) for patient subgroups stratified by high (>median) or low (≤median) expression of the favorable 6-GES and unfavorable 17-GES. Patients with high 6-GES and low 17-GES signature score (green line) exhibited the most favorable outcomes, whereas those with low 6-GES and high 17-GES (orange line) had the poorest outcome. Numbers in parentheses indicate the number of patients in each subgroup. P-values from log-rank tests compare the survival distributions between high and low GES groups. **(D)** The putative predictive value of the 6-GES and 17-GES is presented within the GCB and non-GCB subgroups in the Axi-Cel Arm. Kaplan–Meier plots showing PFS in patients treated with axi-cel, stratified by cell-of-origin (GCB or non-GCB) and 6-GES or 17-GES high vs. low groups. The left panel displays PFS (per central review) for patients classified using a six-gene GES, whereas the right panel shows PFS for those classified by a 17-gene GES. Within each GES, patients are divided into high- and low-risk groups separately for GCB and non-GCB subtypes. P-values from log-rank tests compare the survival distributions between high and low GES groups. Abbreviations: GCB, germinal center B-cell-like; GES, gene expression signature.

The association of the 6-GES and 17-GES with efficacy readouts was evaluated by stratifying patients into “high” and “low” groups based on the median signature scores. Kaplan–Meier curves illustrate the significant associations (descriptive P< 0.05) between the 6-GES or 17-GES and time-to-event efficacy readouts (DOR, EFS, or PFS; **Figure 1B**). High expression of the favorable 6-GES correlated with improved DOR (HR: 0.29, 95% CI: 0.17–0.52), EFS (HR: 0.27, 95% CI: 0.16–0.44), and PFS (HR: 0.27, 95% CI: 0.16–0.46) in axi-cel-treated patients, whereas high levels of the unfavorable 17-GES negatively correlated with DOR (HR: 7.59, 95% CI: 3.95–14.60), EFS (HR: 6.12, 95% CI: 3.57–10.50), and PFS (HR: 7.47, 95% CI: 4.11–13.57) (**Figure 1B**; P< 0.0001 for all readouts). These associations were specific to the axi-cel arm of ZUMA-7 and not observed in patients receiving standard-of-care (SOC) treatment (**Figure 1C**), underlying the potential predictive value of these signatures in the axi-cel treatment setting. These signatures also strongly associated with PFS per investigator assessment (**Figure 2A**).

**Figure 2A** presents the univariate associative analysis of 6-GES or 17-GES or each individual transcripts or predefined GES from the NanoString IO-360 panel with PFS outcome (per investigator), following axi-cel treatment in ZUMA-7: as shown, 6-GES and 17-GES outperformed all individual genes and predefined GES for their association with PFS (**Figure 2A**). Notably, of the 6 or 17 transcripts identified by the multivariate analysis based on the non-zero coefficient around DOR, EFS, and PFS readouts, only CD19, CD45RA, ESR1, and DUSP5 were significantly associated with PFS in univariate analysis, consistent with the notion that 6-GES and 17-GES can carry predictive value not recapitulated by individual transcripts. **Figure 2** also shows that there could be added value in combining 6-GES and 17-GES: the two GES significantly (P = 0.01), but modestly (Spearman R = −0.16) correlated (negatively) with each other (**Figure 2B**; **Supplementary Figure 3**), and subgrouping based on a combination of high and low 6-GES and 17-GES could further improve risk stratification of axi-cel patients (**Figure 2C**). **Supplementary Figure 1** shows a correlation matrix of the 6-GES and 17-GES with the predefined IO360 GES (from NanoString) and the stromal and immune-suppressive index (SII), previously reported (3). This shows that 6-GES correlated with the favorable B-cell signature and represents a less immune infiltrated TME, whereas 17-GES correlated with a more complex and immune-infiltrated TME, including enrichment of the SII (3).

### Predictive value of novel GESs in GCB and non−GCB subgroups in the Axi−Cel Arm

The predictive value of 6-GES and 17-GES was further evaluated in patients treated with axi-cel, stratified by the cell-of-origin subtype. As shown in **Figure 2D**, the Kaplan–Meier curves illustrate the PFS based on GES and cell-of-origin (COO) classification, defined as GCB vs. non-GCB (ABC + unclassified) (3). In the germinal center B-cell (GCB) subgroup, patients with a high 6-GES had significantly (P< 0.05) longer PFS compared to those with a low 6-GES. The 17-GES was also strongly associated with PFS in the GCB subgroup, with patients having a low 17-GES experiencing better PFS outcomes. Among non-GCB patients, the 17-GES remained associated with PFS, whereas the 6-GES did not significantly stratify patients in this subgroup, albeit there was a trend (P=0.088) with a clear separation between the KM curves. Consistently, **Supplementary Figure 2** shows that the 6-GES and 17-GES do not associate with COO (GCB vs. non-GCB; **Supplementary Figures 2A, B**). The 6-GES is also not associated with double-/triple-hit status (high-grade B-cell lymphoma (HGBL)), double-expressor (overexpression of MYC and BCL2 proteins), or MYC rearrangement status, whereas the 17-GES was lower in the HGBL and MYC rearrangement subgroup, compared to LBCL not otherwise specified (not applicable indicates LBCL not belonging to the other molecular subgroups; **Supplementary Figures 2C, D**).

### Technical replication of results using a ZUMA-7 RNA−Seq dataset

To corroborate the association between the favorable 6-GES and unfavorable 17-GES with axi-cel efficacy in ZUMA-7, we analyzed an RNA-seq dataset derived from a subset of ZUMA-7 tumor samples. A total of 21 transcripts overlapped between RNA-seq and NanoString quantification; CD45RA and CD45RO (CD45 isoforms) were excluded from the RNA-Seq analyses because RNA-seq transcript quantification was performed at the gene level to retain data robustness. Hence, in the context of RNA-Seq datasets, a 5-GES and a 16-GES were generated, consisting of the 6-GES minus the CD45RA transcript and 17-GES minus the CD45RO transcript. Spearman correlations across NanoString- and RNA-Seq-derived GESs is shown in **Supplementary Figure 3**. The 5-GES and the 16-GES generated from RNA-Seq strongly associated with efficacy endpoints, including DOR, EFS, and PFS (**Supplementary Figure 4**). Patients with a high 5-GES value exhibited improved DOR, EFS, and PFS compared to those with a low 5-GES value (P< 0.05). Conversely, patients with a high 16-GES score demonstrated inferior outcomes across all three endpoints relative to those with a low 16-GES score.

The ability to recapitulate these findings in a dataset generated through an orthogonal technology reinforces the robustness of the favorable 6-GES (or 5-GES for RNA-Seq) and unfavorable 17-GES (or 16-GES for RNA-Seq) as potential predictive biomarkers of outcome following axi-cel therapy in R/R LBCL patients in the second-line setting.

### Lack of association with PFS in the first-line setting (with R−CHOP/R-CHOP-like treatment)

Next, we evaluated the association of the 5-GES (6-GES without CD45RA) or 16-GES (17-GES without CD45RO) with PFS outcome in two publicly available LBCL RNA-Seq datasets where patients were treated with 1^st^ Line R-CHOP or R-CHOP-like therapy and have corresponding PFS data; for this, we utilized the datasets from Schmitz et al. and Reddy et al. (7, 8). The dataset from Schmitz et al. (8) included 229 patients, whereas the dataset from Reddy et al. included 522 patients (7).

The 5-GES and 16-GES while being associated with outcome to axi-cel in ZUMA-7 (P< 0.05; **Supplementary Figure 4**) did not show an association with PFS to 1^st^ Line R-CHOP/R-CHOP like (**Supplementary Figure 5**), indicating their predictive (specific to CAR T-cell therapy), rather than prognostic, value.

### Expression of 6-GES and 17-GES at disease progression post axi-cel treatment

**Figure 3:**
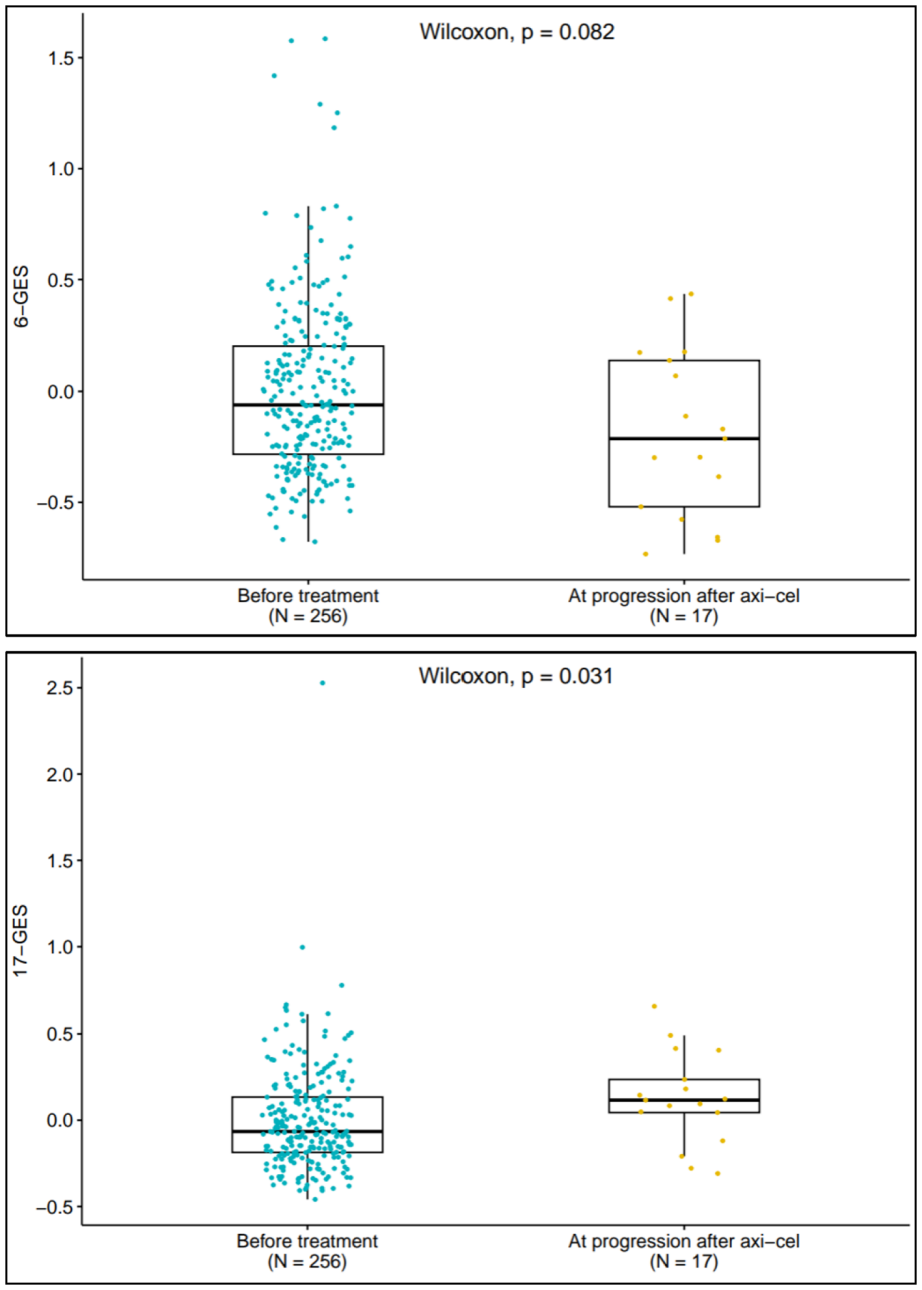
6-GES and 17-GES at disease progression following Axi-Cel treatment. Box plots displaying the expression scores of 6-GES (left panel) and 17-GES (right panel) in tumors collected before ZUMA-7 treatments, both arms (n=256) and in tumors collected at the time of disease progression after axi-cel (n=17). P-value for two-group comparison is calculated by using Wilcoxon rank-sum test.

The 6-GES and 17-GES were analyzed in evaluable patients (available tumor biopsy) who progressed following axi-cel treatment (n=17). The boxplots in **Figure 3** depict the distribution of 6-GES and 17-GES scores before treatment (n=256) and at disease progression following axi-cel treatment. At the time of progression, the favorable 6-GES score was numerically lower, albeit not significant by descriptive statistics (P=0.082), whereas the unfavorable 17-GES was significantly increased (P=0.031), compared to pretreatment levels. These findings corroborate that the 6-GES and 17-GES are likely representative of response and resistance mechanisms and further support previous observations (9) that the TME and the biomarkers associated with outcome can change through lines of therapy.

## Discussion

Axi-cel and other CAR T cell therapies have been delivering transformative results across a number of hematological malignancies in the r/r setting, including diffuse large B-cell lymphoma, follicular lymphoma, mantle cell lymphoma, acute lymphoblastic leukemia, and multiple myeloma (10). CAR T cells are also moving into earlier lines of therapy (11–13), whereas bispecific antibodies are becoming increasingly available options (14). However, there is still an incomplete understanding of the mechanisms of resistance to these therapies and a scarcity of reports around possible predictive biomarkers that could support risk stratification and decisions around sequencing of these therapies. Recently, gene expression profiling of tumors has shown promise for the identification of factors impacting CAR T-cell therapy in R/R LBCL where B cells, as well as stromal and immunosuppressive gene signatures, are emerging as important and interrelated determinants of durable responses to axi-cel intervention (3, 9).

This study leveraged ZUMA-7, the largest available clinical dataset for evaluating second-line CAR T-cell therapy in R/R LBCL, to conduct transcriptomic analyses that identified two novel gene expression signatures (GESs), herein referred to as 6-GES and 17-GES, potentially predictive of outcome following axi-cel treatment.

The 6-GES is composed of CD19, CD45RA, CCL22, KLRK1, SIGLEC5, and SOX11, and the 17-GES signature is composed of IL18R1, GPC4, KIR3DL2, ITGB8, PSMB5, RPS6KB1, BCL2, TNFSF4, SERPINA9, DUSP5, NBN, GLUD1, ESR1, CD45RO, ARID1A, KLRB1, and SLC16A1. The presence of CD19 in the favorable 6-GES (signature associated with better outcome) is not surprising, and it is consistent with our previous observations where tumor target antigen expression associated with EFS (3). The role of the other five genes in the 6-GES signature is not immediately clear. Piseddu et al. reported that CD11c+ DCs are the exclusive producers of CCL22 in secondary lymphatic organs during homeostasis and that a paracrine signaling from T cells was essential for CCL22 secretion (15); hence, CCL22 might represent a tumor microenvironment where there is close communication between T cells and dendritic cells. KLRK1 could speak for immune infiltration enriched in NK cells, NKT, and other cytotoxic lymphocytes, including CD8 cells (16), whereas SIGLEC5 is a putative adhesion molecule that could serve as a checkpoint for T and other immune cells (17–19), perhaps dampening the inflammation that would otherwise be initiated by the KLRK1+ cells. Dictor et al. previously reported no expression of SOX11 in LBCL (20), and its meaning within the 6-GES is unclear, as it is for CD45RA. Overall, the 6-GES might capture lymphomas with abundant antigen expression and adhesion molecules and an immune infiltration characterized prevalently by cytotoxic lymphocytes (T and NK cells) and DCs; the latter might be primed in support of durable responses once CAR T cells infiltrate the tumor. In fact, activity of non-CAR T cells in LBCL has been postulated to strongly contribute to the dept of the response post axi-cel treatment (21) through antigen spreading (22).

On the other hand, the unfavorable 17-GES appear to represent a more complex TME (compared to the tumors with elevated 6-GES) with higher and more heterogenous immune infiltration and concurrent inflammation (e.g., IL18R1; KLRB1 (CD161); KIR3DL2; TNFSF4 (OX40L); DUSP5) (23–27), and activation of immune-escape mechanisms such as upregulation of genes involved in repair of damaged DNA or chromatin remodeling (NBN and ARID1A (28, 29);, inhibition of apoptosis (BCL2 (30, 31);), survival of aggressive tumors (RPS6KB1 (32);), immuno-suppressive myeloid cells (PSMB5 (33);), and a metabolically restrictive TME (e.g., SLC16A1 and GLUD1 (34, 35);). Some of the above might inform therapeutic intervention. For instance, modulation of BCL-2 (36) or targeting of the IL18R1 could improve outcome with CAR T cells. Notably, in ZUMA-7, we found that IL-18 (ligand for IL18R1) gene expression was associated (P< 0.05) with better PFS in univariate analysis. Consistently, CAR T cells armored to secrete IL-18 have shown encouraging clinical results in r/r LBCL, even after prior CAR T-cell treatment (37).

The role of the protease inhibitor SERPINA9 within the 17-GES is unclear and intriguing because SERPINA9 has been previously associated with good prognosis in the context of 1^st^ LBCL (38, 39). Previously, we reported that HGBL and Cell Of Origin status (GCB vs. non-GCB), which are associated with poorer outcome in the setting of first-line R-CHOP therapy (40, 41), are not associated with outcome to axi-cel in the second-line setting (3). Here, we report that the 6-GES and the 17-GES, which associated with DOR, EFS, and PFS following axi-cel treatment, did not associate with DOR, EFS, or PFS following second-line standard-of-care treatment in ZUMA-7 or with PFS after first-line therapy (R-CHOP or -CHOP like) in online datasets, presenting these GESs as putatively predictive to outcome following CAR T-cell therapy in r/r LBCL. These two signatures also outperformed all previous tumor biomarkers associated with disease progression after axi-cel treatment, including the B-cell signature from NanoString (3).

All of the above, jointly with the evidence that the TME evolves through lines of therapy (3, 42), underlies the need for deeper understanding of the prognostic vs. predictive value of these biomarkers in the context of multiple therapies and how these biomarkers, and the tumor biology that they represent, evolve trough disease stages and treatments. This shall lead to better risk stratification and development of next-generation CAR T-cell products or combination strategies to overcome resistance.

This study had certain limitations. The analyses herein are exploratory and retrospective. Conclusions drawn from these data will require confirmation in an independent validation cohort. Extrapolation of these findings in the real-world setting or in prospective clinical studies is warranted.

## Data Availability

The data analyzed in this study is subject to the following licenses/restrictions: Kite is committed to sharing clinical trial data with external medical experts and scientific researchers in the interest of advancing public health. As such, Kite shares anonymized individual patient data (IPD) upon request or as required by law and/or regulation. ZUMA-7 nanostring dataset was employed for discovery of the gene expression signatures. The data presented in the study are deposited in the GEO repository, accession number GSE248835. 1^st^ Line R-CHOP or R-CHOP like therapy RNA-seq datasets are publicly available (7, 8). ZUMA-7 RNAseq dataset used for technical validation of the GES discovered from NanoString dataset: patient-level data of the 16-GES and 5-GES, as well as the gene expression counts (TPM) of the individual transcripts the GES are composed of can be found in the **Supplementary Table 2**, jointly with clinical outcomes. Requests to access these datasets should be directed to medinfo@kitepharma.com.

## Supplementary material

Supplementary Figure 1 correlation matrix of the 6-GES, the 17-GES, the pre-defined IO-360 GES and the stromal and immune-suppressive index (SII). Correlation matrix of all nanostring IO360 pre-defined GES(s) and the previously reported stromal and immuno-suppressive index (SII) Vs 6-GES or 17-GES (nanostring dataset), where Spearman R value is represented by scale-coloring, with positive correlations in red and negative correlations in blue. P values< 0.05 are indicated for each correlation with a “*” sign.

Supplementary Figure 2 Correlation of the 6-GES or 17-GES with COO (GCB Vs non-GCB), double/triple hit (HGBL), MYC rearrangement or double-expressor status. The 6-GES **(A, C)** or 17-GES **(B, D)** signature values (calculated from nanostring dataset) are represented based on molecular subgroups of cell of origin (COO; GCB Vs non-GCB; **(A, B)** or Double-expressor, double/triple hit (HGBL) or MYC re-arrangement, compared to LBCL not otherwise classified (Not Applicable = LBCL not belonging to the other molecular subgroups; **(C, D)**. Box plots present all datapoints (dots), with median represented as the horizontal bar, inter-quartile range captured as the box borders, and whiskers representing values within 1.5x IQR above or below the box. P-value for 2-group comparison is calculated by using Wilcoxon rank sum test, as indicated. P-value for > 2 groups comparison is calculated by using Kruskal-Wallis test, as indicated.

Supplementary Figure 3 Correlations between the GES(s). **(A)** Correlation matrix of GES(s) from nanostring or RNAseq, where Spearman R value is represented by scale-coloring, with positive correlations in red and negative correlations in blue; R values are indicated. **(B)** P values pertinent to the Spearman correlation matrix shown in panel A, where the P values are represented in Log scale and indicated.

Supplementary Figure 4 Technical replication of the GE signatures with efficacy in a ZUMA-7 RNA-seq dataset. Kaplan-Meier curves show DOR, EFS, and PFS (per central review) stratified by median scores of the 5-GES (6-GES without CD45RA)* or 16-GES* from ZUMA-7 RNAseq dataset in axi-cel- **(A)** or SOC- **(B)** treated patient groups (two arms of ZUMA-7 study). P-values from log-rank tests compare the survival distributions between high and low GES groups.*CD45RA and CD45RO transcripts were excluded from the RNAseq analyses because RNA-seq transcript quantification was performed at gene level to retain data robustness; hence, the 5-GES and 16-GES were generated, composed of 6-GES or 17-GES without the CD45RA or CD45RO transcript, respectively.

Supplementary Figure 5 5-GES and 16-GES* are not associated with PFS in 1st line setting with R-CHOP/R-CHOP like treatment (online datasets). Kaplan-Meier curves show PFS stratified by median scores of the 5-GES (6-GES without CD45RA)* **(A)** or 16-GES (17-GES without CD45RO)* **(B)** in R-CHOP/R-CHOP-like patients from two RNAseq datasets of 1^st^ Line setting (publicly available from “Schmitz” et al. or “Reddy” et al.). P-values from log-rank tests compare the survival distributions between high and low GES groups. *CD45RA and CD45RO transcripts were excluded from the RNAseq analyses because RNA-seq transcript quantification was performed at gene level to retain data robustness; hence, the 5-GES and 16-GES were generated, composed of 6-GES or 17-GES without the CD45RA or CD45RO transcript, respectively.
