# Supplementary figures and images for: Tumor gene expression signatures associated with outcome in large B−cell lymphoma treated with CD19-directed CAR T−cell therapy (axicabtagene ciloleucel)
Source: Front Oncol. 2025 Feb 27;15:1519473. doi: 10.3389/fonc.2025.1519473 (PMC11903469; doi:10.3389/fonc.2025.1519473)

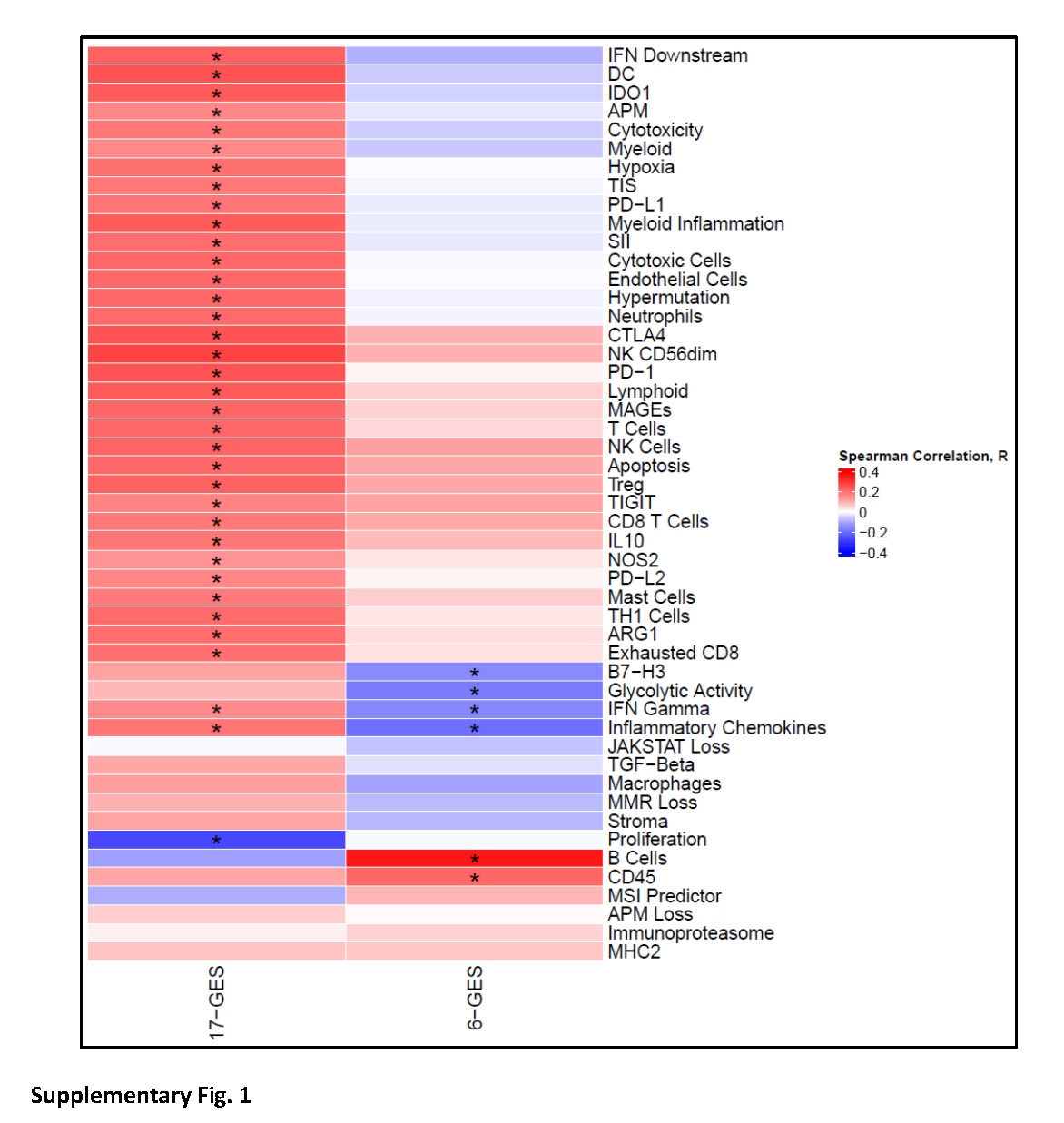

Supplement: Supplementary Figure 1 — correlation matrix of the 6-GES, the 17-GES, the pre-defined IO-360 GES and the stromal and immune-suppressive index (SII). Correlation matrix of all nanostring IO360 pre-defined GES(s) and the previously reported stromal and immuno-suppressive index (SII) Vs 6-GES or 17-GES (nanostring dataset), where Spearman R value is represented by scale-coloring, with positive correlations in red and negative correlations in blue. P values< 0.05 are indicated for each correlation with a “*” sign. [file Image1.tif]

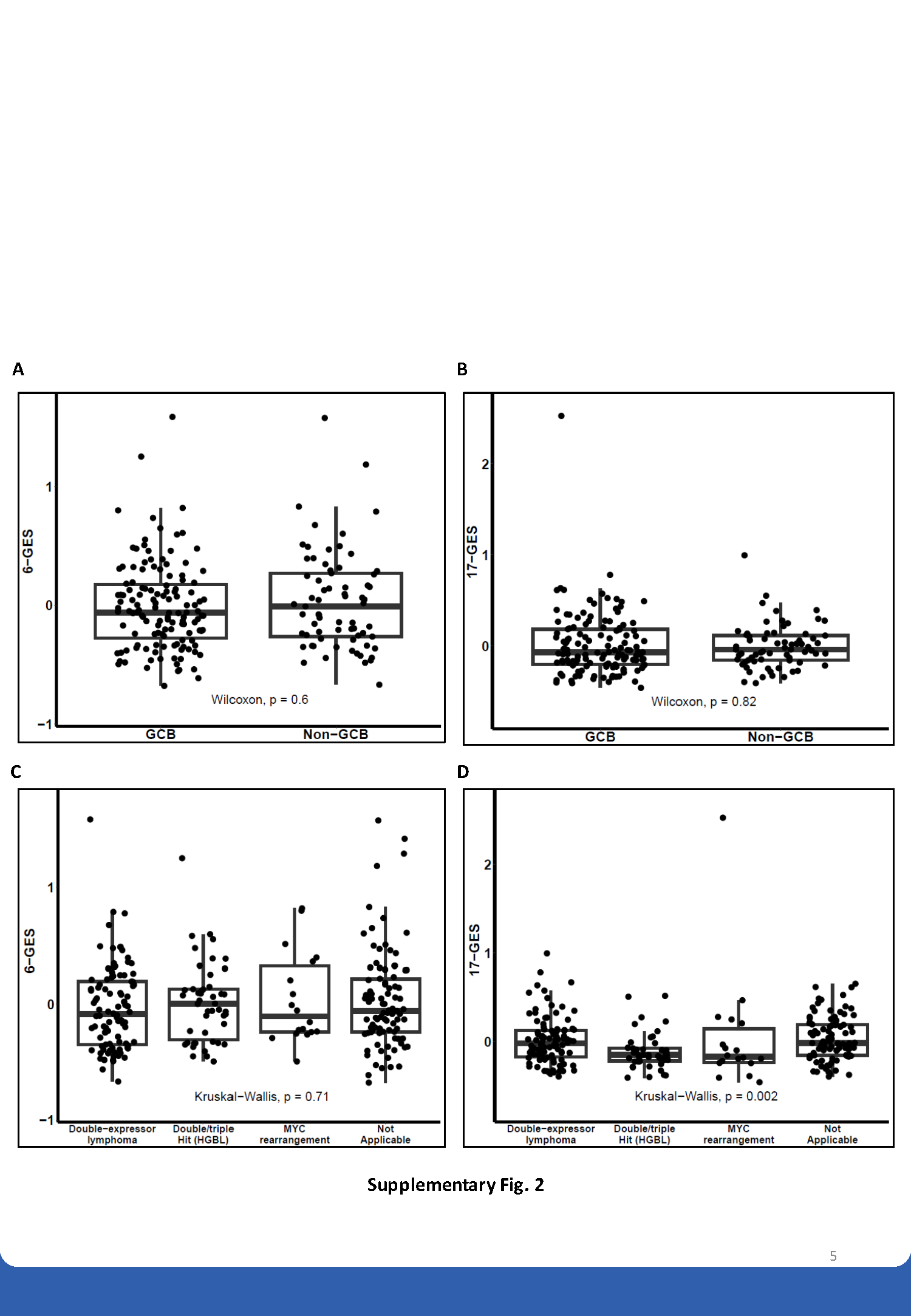

Supplement: Supplementary Figure 2 — Correlation of the 6-GES or 17-GES with COO (GCB Vs non-GCB), double/triple hit (HGBL), MYC rearrangement or double-expressor status. The 6-GES (A, C) or 17-GES (B, D) signature values (calculated from nanostring dataset) are represented based on molecular subgroups of cell of origin (COO; GCB Vs non-GCB; (A, B) or Double-expressor, double/triple hit (HGBL) or MYC re-arrangement, compared to LBCL not otherwise classified (Not Applicable = LBCL not belonging to the other molecular subgroups; (C, D). Box plots present all datapoints (dots), with median represented as the horizontal bar, inter-quartile range captured as the box borders, and whiskers representing values within 1.5x IQR above or below the box. P-value for 2-group comparison is calculated by using Wilcoxon rank sum test, as indicated. P-value for > 2 groups comparison is calculated by using Kruskal-Wallis test, as indicated. [file Image2.tif]

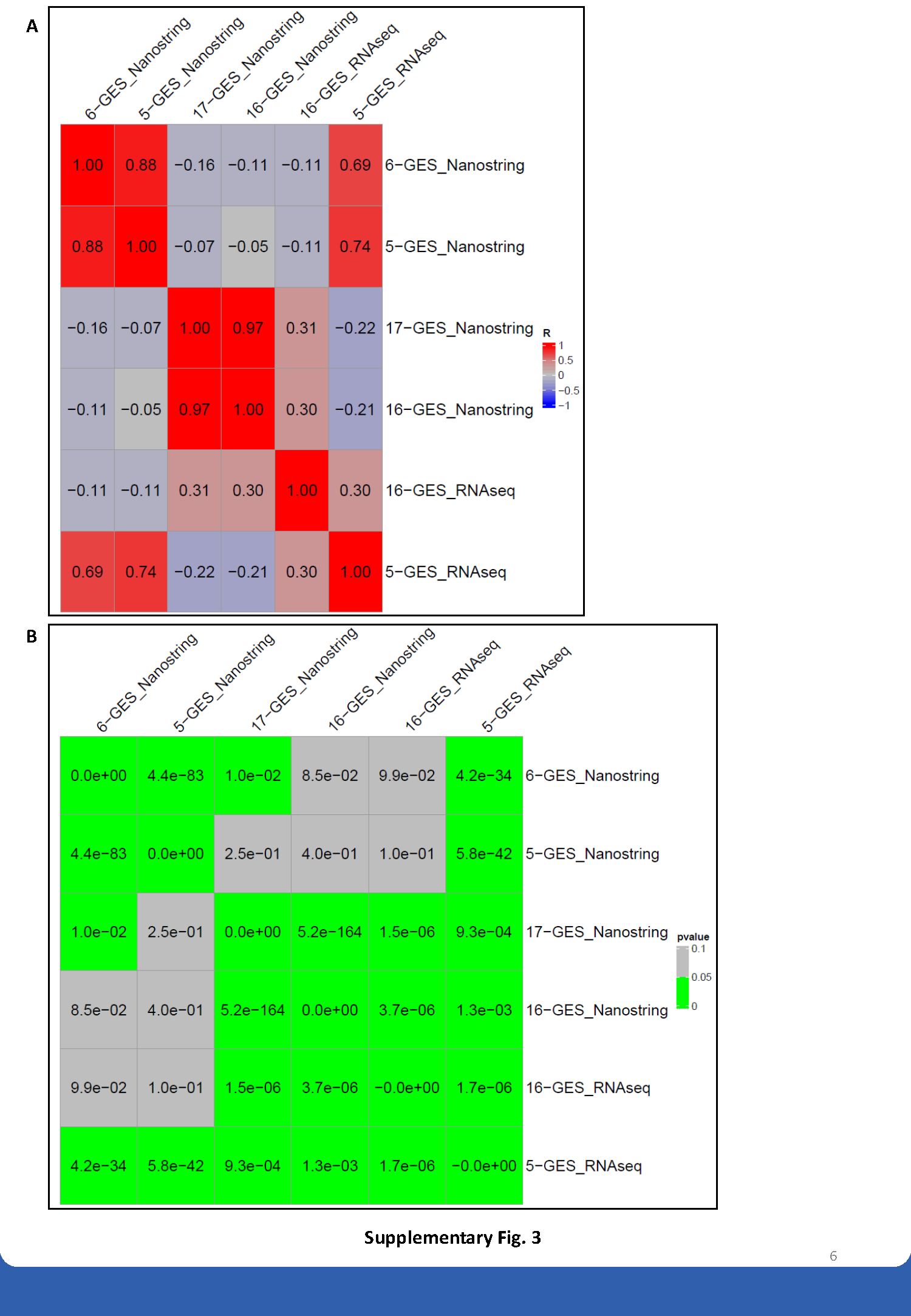

Supplement: Supplementary Figure 3 — Correlations between the GES(s). (A) Correlation matrix of GES(s) from nanostring or RNAseq, where Spearman R value is represented by scale-coloring, with positive correlations in red and negative correlations in blue; R values are indicated. (B) P values pertinent to the Spearman correlation matrix shown in panel A, where the P values are represented in Log scale and indicated. [file Image3.tif]

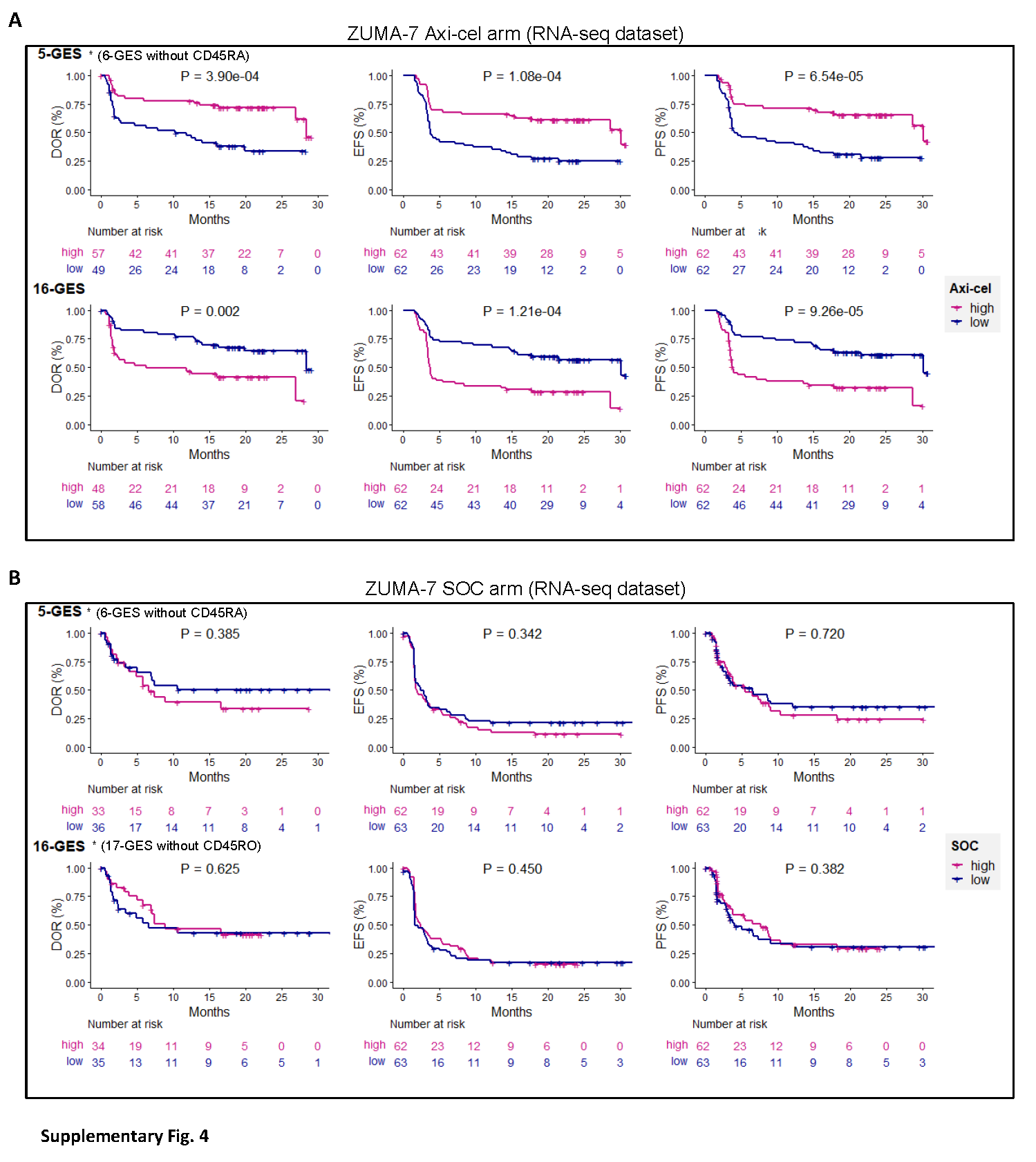

Supplement: Supplementary Figure 4 — Technical replication of the GE signatures with efficacy in a ZUMA-7 RNA-seq dataset. Kaplan-Meier curves show DOR, EFS, and PFS (per central review) stratified by median scores of the 5-GES (6-GES without CD45RA)* or 16-GES* from ZUMA-7 RNAseq dataset in axi-cel- (A) or SOC- (B) treated patient groups (two arms of ZUMA-7 study). P-values from log-rank tests compare the survival distributions between high and low GES groups.*CD45RA and CD45RO transcripts were excluded from the RNAseq analyses because RNA-seq transcript quantification was performed at gene level to retain data robustness; hence, the 5-GES and 16-GES were generated, composed of 6-GES or 17-GES without the CD45RA or CD45RO transcript, respectively. [file Image4.tif]

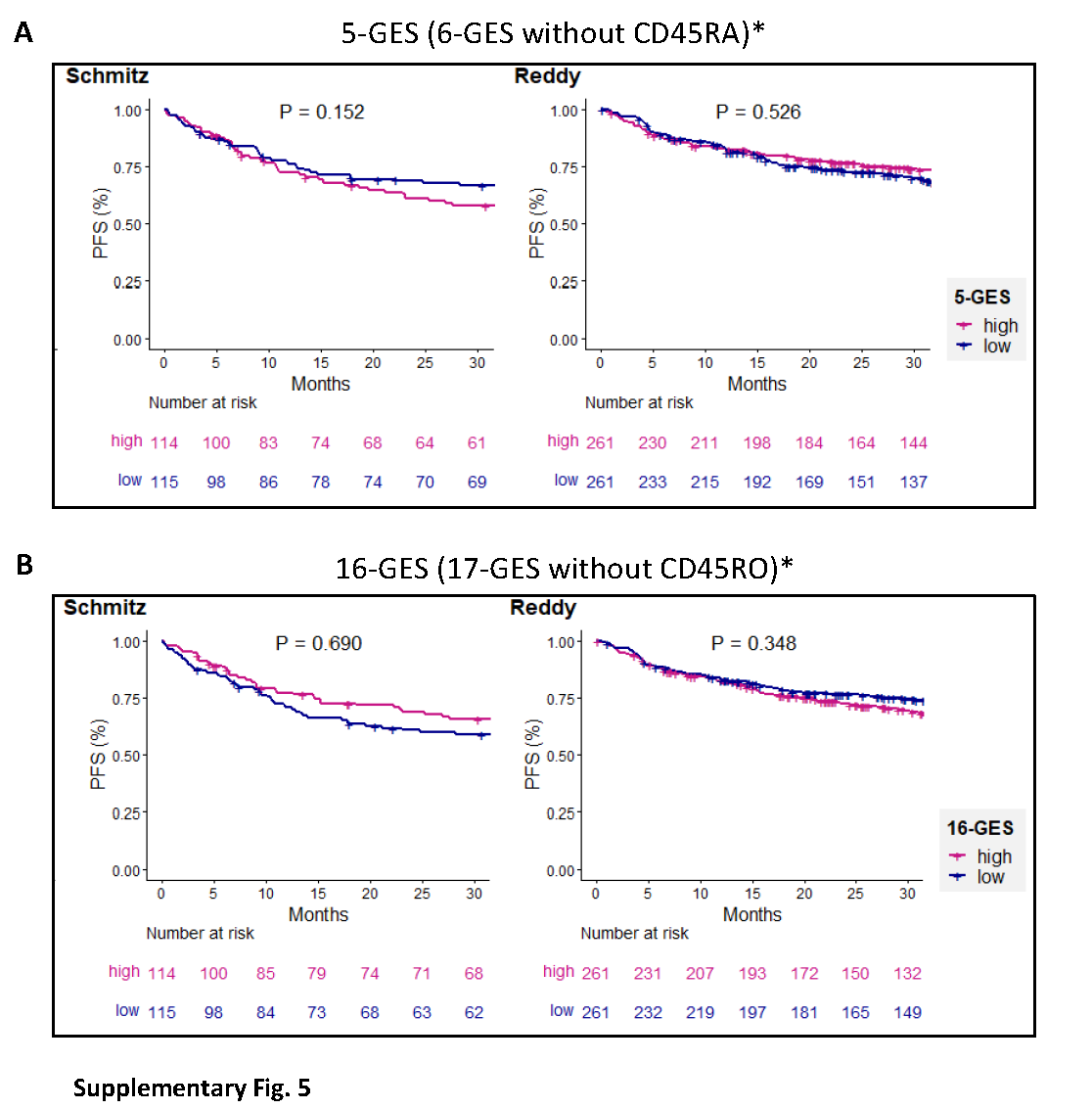

Supplement: Supplementary Figure 5 — 5-GES and 16-GES* are not associated with PFS in 1st line setting with R-CHOP/R-CHOP like treatment (online datasets). Kaplan-Meier curves show PFS stratified by median scores of the 5-GES (6-GES without CD45RA)* (A) or 16-GES (17-GES without CD45RO)* (B) in R-CHOP/R-CHOP-like patients from two RNAseq datasets of 1st Line setting (publicly available from “Schmitz” et al. or “Reddy” et al.). P-values from log-rank tests compare the survival distributions between high and low GES groups. *CD45RA and CD45RO transcripts were excluded from the RNAseq analyses because RNA-seq transcript quantification was performed at gene level to retain data robustness; hence, the 5-GES and 16-GES were generated, composed of 6-GES or 17-GES without the CD45RA or CD45RO transcript, respectively. [file Image5.tif]
