# Supplementary material for: Tumor gene expression signatures associated with outcome in large B−cell lymphoma treated with CD19-directed CAR T−cell therapy (axicabtagene ciloleucel)
Source: Front Oncol. 2025 Feb 27;15:1519473. doi: 10.3389/fonc.2025.1519473 (PMC11903469; doi:10.3389/fonc.2025.1519473)
Supplement: Supplementary file 6 [file DataSheet1.pdf]

DOR	EFS	PFS
KLRK1	KLRK1	KLRK1
IFNGR2	CXCL13	CCL22
IFI16	CCL22	ADORA2A
TWF1	MMP9	SOX11
PARP4	SOX11	ICAM3
ATM	MARCO	MARCO
CCL22	HRAS	FGF9
SOX11	LY9	DKK1
ICAM3	WNT2	TNFRSF17
WNT2	DKK1	BTLA
BRIP1	H2AFX	WNT7B
NRAS	HLA-DQB1	BID
WDR55	BTLA	NRAS
CD48	FCN1	CDK2
CD19	BID	CD19
CD45RA	CD44	CD45RA
RASGRF1	OLFML2B	HLA-DQA2
ARG2	CDK2	HMGB1
CCL7	RRM2	ARG2
CTAG1B	CD19	HLA-DOB
TCL1A	CD45RA	ISY1
FAS	HLA-DQA2	MS4A1
MICB	TLR4	DPP4
ICOSLG	HMGB1	MS4A6A
SIGLEC5	HLA-A	TAP2
PFKFB3	CCND3	MICB
	MS4A1	TNFRSF8
	IRF8	ICOSLG
	SIGLEC5	SIGLEC5

DOR	EFS	PFS
NBN	NBN	NBN
FANCA	NCAM1	NCAM1
CD45RO	CD45RO	NFKB1
CD8B	IL1A	CD45RO
CES3	C7	CD8B
IL1A	ARID1A	IL1B
IL1B	GPC4	S1PR2
HERC6	KIR3DL2	IL33
C7	CD80	ARID1A
S1PR2	IRF4	GPC4
ARID1A	BLM	KIR3DL2
GPC4	PLA1A	CD80
KIR3DL2	MME	CD45RB
PNOC	SMAP1	BLM
SOX10	WNT11	MME
TNFSF4	BAMBI	WNT11
BCL2	TNFSF4	BAMBI
MAML3	BCL2	KLRD1
DUSP5	SOX2	TNFSF4
RPS6KB1	DUSP5	HES1
GLUD1	RPS6KB1	BCL2
PDGFA	IKBKG	DTX4
GBP1	GLUD1	SOX2
DEPTOR	BAX	DUSP5
EPCAM	CD69	RPS6KB1
IL18R1	SFRP4	IKBKG
INHBA	CCL20	GLUD1
GZMB	IL18R1	PDGFA
AKT1	INHBA	BAX
LIF	SFXN1	THBS1
CX3CR1	LIF	EPCAM
CCL19	CD209	ASB13
PSMB5	PSMB5	CCL20
ESR1	ESR1	IL18R1
SLC16A1	SLC16A1	SFXN1
MGMT	MGMT	CCL19
ITGB8	TNFRSF25	LOXL2
KLRB1	ITPK1	PSMB5
PSMB10	ITGB8	ZEB1
NOS2	KLRB1	ESR1
SERPINA9	RIPK2	SBNO2
	ENO1	SLC16A1
	NOS2	TNFRSF25
	SERPINA9	ITPK1
		ITGB8
		KLRB1
		BBC3
		SERPINA9
